# Thalassemias in South Asia: clinical lessons learnt from Bangladesh

**DOI:** 10.1186/s13023-017-0643-z

**Published:** 2017-05-18

**Authors:** Mohammad Sorowar Hossain, Enayetur Raheem, Tanvira Afroze Sultana, Shameema Ferdous, Nusrat Nahar, Sazia Islam, Mohammad Arifuzzaman, Mohammad Abdur Razzaque, Rabiul Alam, Sonia Aziz, Hazera Khatun, Abdur Rahim, Manzur Morshed

**Affiliations:** 1 Biomedical Research Foundation, House #7, Apartment# 1A, Road# 1/B, Banani, Chairman Bari, Dhaka-1213, Bangladesh; 2 Faculty of Basic Sciences, Bangladesh University of Health Sciences, Dhaka, Bangladesh; 3 School of Environmental Science and Management, Independent University, Dhaka, Bangladesh; 4 Thalassemia Foundation Hospital, Dhaka, Bangladesh; 5 Trinity College, Dublin, Ireland; 6 Department of Economics & Business, Moravian College, Bethlehem, USA; 7 Saint Michael’s Medical Center, Newark, NJ USA

**Keywords:** Beta thalassemia, Hemoglobinopathies, Thalassemia trait, Beta thalassemia major, HbE beta thalassemia, Non-transfusion dependent thalassemia, Transfusion dependent thalassemia, Bangladesh

## Abstract

Thalassemias are emerging as a global public health concern. Due to remarkable success in the reduction of childhood mortality by controlling infectious diseases in developing countries, thalassemias are likely to be a major public health concern in the coming decades in South Asia. Despite the fact that Bangladesh lies in the world’s thalassemia belt, the information on different aspects (epidemiology, clinical course, mortality, complications and treatment outcomes) of thalassemias is lacking. In this comprehensive review, the aim is to to depict the epidemiological aspects of thalassemias, mutation profile and current treatment and management practices in the country by sharing the experience of dealing with 1178 cases over 2009–2014 time periods in a specialized thalassemia treatment centre. We have also discussed the preventative strategies of thalassemias from the context of Bangladesh which could be effective for other developing countries.

## Background

Inherited hemoglobin disorders are emerging as a global public health concern. An estimated 320,000 babies are born each year with a clinically significant hemoglobin disorder [1]. Nearly 80% of these births occur in developing countries. Most conservative estimates suggest that at least 5.2% of the world population (over 360 million) carry a significant hemoglobin variant [1] and in excess of 100 million beta thalassemia carriers with a global frequency of 1.5% [2]. Homozygous or compound heterozygous states between certain variants can lead to clinical manifestations of hemoglobinopathies.

The inherited beta thalassemias including sickle cell anemia and hemoglobin E (HbE) disorders are the most frequent single gene disorders globally [1]. Thalassemia syndromes are caused by an absence or ineffective synthesis of beta globin chains. Hemoglobinopathies are most prevalent in certain malaria prone parts of the world including Africa, all Mediterranean countries, the Middle East, the Indian subcontinent and Southeast Asia [1, 3]. In each year, over 50,000 new patients are born with a severe form of thalassemia (beta-thalassemia major and HbE beta thalassemia) worldwide. Due to high rate of international migration, thalassemias are spreading to non-endemic parts of the world [2]. In many Asian countries, the most common form of thalassemia results from the coinheritance of beta thalassemia and HbE. In the eastern parts of Indian subcontinent, Bangladesh and other Southeast Asian countries, HbE is the most prevalent hemoglobin variant [4].

South Asia, a hotspot of hemoglobinopathies [2], is home to 23% of the world’s population (approximately 1.7 billion) [5]. Most information on thalassemia in South Asia comes from studies conducted in India. Due to extreme heterogeneity, an uneven frequency of beta thalassemia heterozygote or carrier in the range of 1 and 10% has been reported throughout different parts of India [2]. However, the overall prevalence of beta thalassemia carriers has been estimated to be between 2.78 and 4% in India [6, 7]. This number translates to approximately 30–48 million beta thalassemia carriers in India and approximately 5–12 million carriers in Pakistan with a carrier rate of 5–7% [2, 8].

Bangladesh is one of the most densely populated countries in the world, with a population of over 160 million people. Over 70% of the population live in highly resource-constrained rural areas [5], while most tertiary hospitals are located in big cities, notably in Dhaka, the capital city. Public hospitals are often overcrowded and lack resources (such as specialized and basic medical equipment, healthcare professionals and essential drugs) [9]. On the contrary, some private clinics and hospitals are relatively resourceful but these are not accessible to the general population due to the associated costs. The treatment drop-out rate among a population plagued by poverty is expected to be very high, and is presumably driven by lack of access, either due to lack of awareness or income of patients seeking care on the demand side, or inadequate expertise, facilities, knowledge, and infrastructure from the supply side of health care.

Despite the fact that Bangladesh lies in the world’s thalassemia belt, the information on different aspects (epidemiology, clinical course, mortality, complications and treatment outcomes) of thalassemias is lacking. A recent study has revealed that the higher prevalence of anemia in Bangladesh is not associated with iron deficiency [10]. The nationwide prevalence of anemia (33.1% in children under five years of age and 26% in women) was more than three times higher than that of iron deficiency in children (10.7%) and women (7.1%), suggesting other determining factors for this unexpected scenario [10]. In this context, the role of congenital Hb disorders along with micronutrient (such as dietary iron, vitamin A, folate and Zn) deficiency could explain this phenomenon [10]. A recent study has indicated that about 28% of assessed rural women have beta thalassemia or HbE [11]. Similar findings have been reported for women and children of the thalassemia prone Southeast-Asian country, Cambodia [12, 13].

Because of phenomenal success in the control of infectious diseases in Bangladesh, child mortality has declined by 71% as compared to that observed in the 1990s [14]. Genetic disorders, particularly thalassemias, are therefore likely to be a major public health concern in Bangladesh in the coming decades [15]. In this comprehensive review, the objective is to depict the epidemiological aspects of thalassemias and current management practices in the country by sharing the experience based on a specialized thalassemia treatment centre. In addition, we intend to provide preventative strategies of thalassemias from Bangladesh’s perspective.

## Epidemiology

The information on the prevalence of hemoglobinopathies in Bangladesh is scarce due to lack of population-based data. According to World Health Organization (WHO) estimates, approximately 3% of the population are the carriers of beta-thalassemia and 4% are the carriers of hemoglobin E (HbE) in Bangladesh [7]. However, these estimates must be interpreted with caution since the data was mainly based on studies conducted in 1980, and a small number of non-representative samples obtained from treatment centers were analyzed [3]. The only published report available on the prevalence of thalassemia among (*n* = 735) school children in Bangladesh showed a 4.1% prevalence of the beta-thalassemia trait and a 6.1% prevalence for the HbE trait [16]. The same study revealed the regional variation of beta-thalassemia carriers rangin*g* from 2.9 to 8.1% and 2.4 to 16.5% for HbE carriers. Among tribal children, the prevalence of beta-thalassemia trait was almost identical but HbE was much higher (41.7%). Another study with a small sample size also observed similar (39–47%) prevalence rate of HbE among a tribal population in Bangladesh [17].

Bangladesh shares linguistic and socio-cultural commonalities with East Indian region, particularly West Bengal. By and large, the genetic makeup is also closely related in this part of the world [18] despite different religious backgrounds [19]. Hence, the prevalence of hemoglobinopathies in Bangladesh could be extrapolated from population-based studies conducted in West Bengal. A recent large population-based study (*n* = 50,487) in rural West Bengal revealed the carrier rate of beta-thalassemia and HbE to be 6.61 and 2.78% respectively [20]. Another recent study (*n* = 9990) estimated the frequency of 3.64% for beta thalassemia traits and 3.92% for the HbE trait [6]. Taking all these factors into account, the estimated prevalence of beta-thalassemia carriers could be in the range of 3–6%, and 3–4% for HbE in Bangladesh.

It is a fact that most children with severe forms of thalassemia (such as thalassemia major) usually die under 5 years of age [1] and the average life expectancy of patients suffering from thalassemias is about 30 years [20], particularly in heavily resource constrained countries. Considering these factors, we can extrapolate the overall scenario of thalassemic patients in Bangladesh using data from West Bengal [20]. The number of patients suffering from thalassemias (beta major and HbE beta) with different levels of severity is estimated to be approximately 60,000–70,000 in Bangladesh [3]. With the birth rate of 21.6/1000, it could be estimated that nearly 2500 thalassemia major cases are added every year in Bangladesh [20]. Due to considerable variation of thalassemias even within a population, micro mapping is essential to calculate the actual burden of thalassemia in Bangladesh.

Despite the high prevalence of malaria, contrary to the African region and certain parts of India, sickle cell hemoglobin (HbS) is almost non-existent in Bangladesh. Although there has been evidence of a strong association in geographical distribution between malaria and HbS, the relationship was found to be strong in only Africa but not in the Americas or in Asia [21]. In India, HbS variant is mostly confined to the hilly areas and Western region [22]. Due to non-existence of confirmatory diagnostic test (such as sickle solubility test) in most diagnostic centres, it appears that some cases of HbS could be misdiagnosed as HbD beta thalassemia, which is relatively a common form of hemoglobinopathy in Bangladesh and India.

## Mutation profile of thalassemia in Bangladesh

The spectrum of mutations varies across different geographical regions and cultures. Hence, the regional mutation profiling is essential to undertake any strategies (e.g. genetic counseling, prenatal diagnosis) to deal with thalassemias. Different mutations are associated with different types of thalassemias that influence the severity of the diseases. Mutations in globin genes (α or β) affect the synthesis of the globin chain that lead to incomplete erythropoiesis. The diagnosis of alpha thalassemia is often difficult and most cases (approximately 90%) remain as carrier among the population of the Indian Subcontinent. Therefore, alpha thalassemia is beyond the scope of our present discussion [23]. More than 400 mutations or alleles have been reported for beta thalassemia [24].

A meta-analysis comprising 8505 alleles among the Indian population has revealed that five mutations [IVSI-5(G > C), IVSI-1(G > T), 619-bp del, Codon 41/42(-TCTT) and Codon 8/9(G)] accounts for 90% of all beta-globin mutation [25]. IVS I-5 (G > C) is the most prevalent β-thalassemia mutation in South Asia but the frequency varies ranging from 36.5% in Pakistan, 56.3% in India and to 64.6% in Sri Lanka [25].

Despite a large number of thalassemic carriers in Bangladesh, the genetic basis of thalassemia is largely unknown. Virtually no molecular diagnostic service centres are available in the country to pinpoint the mutations in the beta globin gene. To the best of our knowledge, there are only two reports exist on the mutation status of thalassemia in Bangladesh. A recent study (*n* = 256) showed prevalence of the five most common mutations including IVSI-5(G > C), Codon 41/42(-TCTT), Codon 8/9(G) codon 15 (G > A) and codon 30 (G > C), where IVSI-5 was found to be the most common in Bangladeshi patients (39.1%) [26]. In another study (*n* = 16), IVS-I-5 (G > C) accounted for 56.25% [27]. As expected and discussed earlier, the mutation profile (five common mutations) of Eastern India (West Bengal) was found to be similar to that of Bangladesh, IVS I-5(G > C) being the highest form of mutation (71.4%), with Codon 30(G > C) and Codon 15(G > A) the second and third most common alleles [28]. The 619 bp deletion mutation is less frequent in Bangladesh (0.8%) and Eastern India [25, 26].

## Management practice of thalassemia in Bangladesh

Standard thalassemia management comprises of a multidisciplinary approach involving an array of specialties including pediatric hematology, pediatrics, transfusion medicine, endocrinology, cardiology, dentistry, dieticians, psychology, psychiatry, social work along with a robust blood bank system and infrastructure [29]. In developing countries like Bangladesh, these multidisciplinary expertise and support facilities are not usually available in most public hospitals and private clinics. In addition, overall health awareness is very poor among the general population in Bangladesh and there is no organized patient referral system. As a consequence of inadequate access to healthcare, a significant proportion of the thalassemic patients might die even without knowing their disease conditions. There is no national policy or national health insurance system regarding thalassemia prevention in Bangladesh.

To portray the overall current scenario of thalassemia management practice in Bangladesh, the experiences obtained from Thalassemia Foundation Hospital (TFH) are presented in this article.

### Patient and clinical set up of TFH

Our study centre is one of the two specialized hospitals in the country that solely deal with thalassemia patients. TFH is located in Dhaka, the capital city of Bangladesh. This day-care service centre was established by thalassemia support group and families of the patients. Initially, it was a small discussion group to exchange up-to-date information on thalassemia management and the problems faced by thalassemia patients and their families. The number of patients grew substantially over next couple of years. The unavailability of iron chelator medicines and need of a convenient transfusion facility were the major problems raised by the families. To address these problems, TFH started its journey in 2008 to provide day-care services including blood transfusion, expert consultation, medicines and laboratory tests from a single center. The hospital is currently managed by two senior hematologists, one transfusion medicine consultant, and a team of 15 doctors, nurses and support staff.

In this study, patients attending THF from 1/2009 to 12/2014 were included. This hospital-based retrospective study was ethically approved by the Ethical Review Committee of Bangladesh University of Health Sciences (Memo No: BUHS/ERC/16/031). Charts of the patients were reviewed. After registration at the hospital each patient was provided a unique ID to maintain further documentation. At first visit to the center, detailed history, physical examination findings, height and weight, were recorded. CBC, Hb electrophoresis (preferably at the time of diagnosis and prior to transfusion), basic metabolic panel, liver functions tests, and baseline iron status were obtained. At initial visit, patients were assessed for the transfusion requirement. Patients were observed for 4 weeks and were followed up for clinical symptoms and Hb. Patients stable after this period were further monitored to determine the steady state Hb and correlation of Hb with clinical symptoms. This was to categorize the patients clinically into thalassemia intermedia and to determine transfusion trigger. Patients were followed up every 4–6 weeks for quality of life (QOL), worsening organomegaly and growth failure. Transfusion dependent patients were monitored for iron loading and medication side effects. Hydroxyurea was used in thalassemia intermedia patients and were followed up for QOL and Hb increment.

Over a 5-year period (2009–2014), a total of 1594 thalassemia patients were served by TFH out of which 1178 complete cases were analyzed with a male to female ratio of 1.26. All cases of thalassemias were diagnosed using conventional electrophoresis method. Approximately 77.3% of the patients were diagnosed as HbE beta thalassemia, while nearly 15% were beta thalassemia major. About 91% patients (*n* = 971) required blood transfusion, where approximately 66.9% of them were transfusion-dependent thalassemia (TDT) patients and 24.3% were non-transfusion dependent thalassemia (NTDT) (Table 1). About 41.1% of TDT patients required blood transfusion every 2–4 weeks. Due to incomplete medical record, the transfusion history was missing for 115 diagnosed cases (approximately 9.7% of all cases).

**Table 1:** Pattern of thalassemia and transfusion practice in Bangladesh

Diseases types	n (%)	Median age (year) at diagnosis	Transfusion status #/n (%)		
			TDT	NTDT	Not required
Hb-E-beta thalassemia	910 (77.25)	3.5	522/840 (62.14)	238/840 (28.33)	80/840 (9.52)
Beta thalassemia major	173 (14.69)	0.58	172/173 (99.42)	1/173 (0.58)	0/173 (0)
Beta thalassemia trait	64 (5.43)	27.5	8/26 (30.77)	14/26 (53.85)	4/26 (15.38)
Hb E disease	12 (1.02)	9	3/11 (27.27)	3/11 (27.27)	5/11 (45.45)
Hb-E trait	14 (1.19)	26	2/8 (25)	3/8 (37.50)	3/8 (37.50)
Others (H, Punjab D etc.)	5 (0.42)	4	5/5 (100)	0/5 (0)	0/5 (0)
Total	1178		712/1063 (66.98)	259/1063 (24.36)	72/1063 (8.66)

*NTDT*, non-transfusion dependent thalassemia; *TDT*, transfusion dependent thalassemia

### Transfusion practice

Thalassemia carriers are healthy and do not require blood transfusion. Non-transfusion dependent thalassemias generally include HbE beta thalassemia and beta thalassemia intermedia that do not require regular blood transfusions for survival [30]. However, in TFH, we found some diagnosed carrier patients admitted as TDT (10 cases) and NTDT (17 cases). Infection and iron deficiency, particularly in reproductive age females, could compound the pre-existing anemia although these patients are not actually transfusion dependent. This could be due to misdiagnosis and/or lack of awareness among the clinicians in Bangladesh.

Thalassemia intermedia (TI) is defined as a group of patients with beta thalassemia characterized by diverse clinical severity between transfusion dependent thalassemia major and mild symptoms of beta thalassemia trait. Most TI patients are homozygous and compound heterozygous for beta thalassemia [31]. In Southeast Asia including the Indian subcontinent, the most common form of severe thalassemias results from the coinheritance of HbE and beta trait. Based on clinical severity, HbE beta thalassemia could be classified into three categories: mild (15% cases), moderately severe (majority of HbE beta thalassemia cases) and severe. Up to 50% of all patients with HbE beta thalassemia represent clinical manifestations similar to those of beta thalassemia major [32].

Due to extensive clinical diversity, the management of NTDT is often challenging. Diagnosis and management of NTDT mainly depend on clinical observations. In our study, over 62% of HbE beta thalassemia patients were treated as TDT while about 28% were NTDT (Table 1). This unexpected higher proportion of transfusion dependent HbE beta thalassemia in Bangladesh might result from inaccurate or misdiagnosis of the severity of different clinical manifestations of thalassemia patients. It could also be attributable to using Hb level to determine need for transfusion in HbE beta patients as opposed to using other criteria including growth failure, delayed puberty, splenomegaly, tendency to thrombosis and pulmonary hypertension [33]. A complete mutation profile (DNA testing) prior to initiation of treatment is helpful to determine the prognosis, appropriate therapy and family counseling [29].

Increased awareness among clinicians is a prerequisite for proper diagnosis and management of NTDT. Several studies have suggested the limitation of Hb level as a clinical decision indicator for starting transfusion dependent management [30] since there were only minor differences in Hb levels (1.8–2.6 g/dl) between the mildest and most severe forms of HbE beta thalassemia. In addition, some children with HbE beta thalassemia were found to adapt to lower levels of Hb and managed almost normal life without transfusion [34, 35]. In one study conducted in Sri Lanka, approximately 42% (37/84 cases) of patients with HbE beta thalassemia could be reversed from TDT to NTDT without any deleterious medical conditions, suggesting that many of these patients actually received unnecessary regular blood transfusion therapy [36]. Patients with NTDT sometimes could suffer from severe anemia due to acute infection, therefore, transfusion therapy is not recommended immediately after diagnosis of NTDT [32]. Despite this fact, patients are not dependent on regular transfusions for survival although transfusion therapy may provide significant clinical benefits for some patients if administered properly [30].

Apart from patho-physiological, psychological and the financial burden, the regular arrangement of safe blood is one of the biggest challenges faced by transfusion-dependent families in developing countries. In Bangladesh, 85% of collected blood is contributed by patient’s relatives and friends, while the rest (15%) is donated by voluntary blood donors [37]. Taking this into consideration, before starting transfusion therapy, accurate diagnosis should be a mandatory part of the thalassemia management practice in Bangladesh.

### Infection

TDT thalassemia patients are at risk of developing post-transfusion hepatitis. Among these infections, hepatitis B and C are the most common. Due to lack of effective vaccines against HCV and inadequate infection control strategies, HCV is considered as major public problem in low to middle-income countries [38]. Approximately 180.5 million people are infected by HCV in the world, of which 54.4 million is in South Asia [39]. A number of studies have reported the higher prevalence of HCV among multi-transfused thalassemia patients, ranging from 3 to 67.3% [40–43]. Increased risk of HCV infection in β-thalassemia patients is mainly associated with median age, duration, and mean amount of blood transfused. No HIV cases were detected in TFH. In case of HBV, 6 cases were positive among 523 tested cases who had blood transfusion. In the present study, 28.3% of the tested cases (*n* = 247) who underwent multiple transfusions were found to be HCV positive. Another previous study conducted in Bangladesh also observed higher positive cases of HCV among multi-transfused thalassemia patients [28].

### Iron chelation and hydroxyurea therapy

In our study, approximately 43% of the patients (*n* = 972) with multiple blood transfusions were treated with iron chelators to remove excess iron from the body. Deferiprone was the most commonly used iron chelator (*n* = 481) followed by Deferasirox (*n* = 199) and Desferal (*n* = 91). Hydroxyurea therapy was given to nearly 43% of the patients (*n* = 972) who underwent transfusions regularly or occasionally to increase foetal hemoglobin and reduce ineffective erythropoiesis.

### Treatment cost of thalassemia in Bangladesh

The cost of treatment varies according to age, body weight and severity of the disease. The most conservative direct medical cost ranges from BDT 127,000 (USD 1632; USD 1 = BDT 78) to BDT 309,000 (USD 3960) per year (Table 2). There is neither a national insurance system nor subsidized or free treatment from the government health facilities. It is expected that patients must pay for their treatment and it is difficult for most of the families to afford proper treatment. Over 72% of the patients’ (*n* = 448) monthly household income was between BDT 10,000 (USD128) to BDT 20,000 (USD 256), suggesting a huge economic burden that could render seeking treatment for most thalassemia patients unviable in Bangladesh.

**Table 2:** Conservative estimate of treatment cost at Thalassemia Foundation Hospital, Bangladesh

Requirements	Cost (USD)		
	1–10 years	11–20 years	21–30 years
Blood Transfusion plus filter/month	32 (1 unit)	65 (2 units)	96 (3 units)
Iron chelation (Desferrioxamine/deferasirox)	65 (25 vials)	130 (50 vials)	195 (75 vials)
Hospital care 1 day/month	26	26	26
Lab tests/month	13	13	13
Total cost/month	136	234	330
Total cost/year	1632	2808	3960

### Future directions and recommendations

Thalassemias impose a significant burden on healthcare systems in endemic regions since the lifetime management cost of thalassemia is beyond the capacity of resource-constrained countries. It is estimated that only 12% of patients with transfusion-dependent thalassemia are properly transfused and of those less than 40% have access to adequate iron chelation [1].

Due to increased life expectancy and slowing population growth, Bangladesh is now experiencing the double burden of communicable and noncommunicable diseases (NCDs) [9]. Currently, in Bangladesh, NCDs account for 59% of total deaths (WHO), which in turn, has created a strain on the existing healthcare system in country. The government of Bangladesh spends only US $26.60 per capita for healthcare services [44]. The management of the disease is multifaceted and expensive. There is no cure for thalassemia except for allogeneic BMT in a select group of patients which again is a very expensive treatment option. The management of this disease is also very costly. As mentioned earlier, an average Bangladeshi family has to spend more than their monthly household income for a thalassemia major patient. However, the prevention of thalassemia is cost effective; at least four times less expensive than treating thalassemia based on a study conducted in Israel [45]. Prevention is therefore likely to be the most viable strategy to reduce the burden of the thalassemia patients on families and to manage a sustainable healthcare system.

### Pre-marital screening

A primary preventive program is based on the carrier [heterozygous] detection and counselling to discourage marriage between carriers. Overall, premarital screening for thalassemia and other preventable genetic diseases are widespread in many parts of the world [46]. The success of mandatory premarital screening with genetic counseling was only effective in reducing beta thalassemia births in some Middle Eastern countries (Iran, Turkey) because of widespread awareness, screening timing and access to prenatal diagnosis (PND) and the option of therapeutic abortion [47]. In addition, Taiwan adopted a national screening program in 1993 to manage the spread of thalassemia which enjoyed considerable success, with less than three per year of thalassemia births in last 10 years [48]. Prevention via pre-marital and genetic screening is arguably the best approach to prevent thalassemias considering socio-religious issues, among other factors. However, the success of mandatory premarital screening with genetic counseling was only effective in reducing beta thalassemia births in some Middle Eastern countries (Iran, Turkey) because of widespread awareness, screening timing and access to prenatal diagnosis (PND), and the option of therapeutic abortion [47]. In conservative societies, marriage is a very complex social phenomenon where couples are usually selected based on strong personal preference as well as traditional reasons. Depending on the thalassemia carrier status, if a planned marriage is called off, it may cause social embarrassment or stigmatization to the young couples and their families.

### Target screening approaches

Due to inadequate healthcare access as well as infrastructure and financial constraints, the antenatal screening in pregnant women is not a practically feasible approach since the vast majority of the women, particularly in resource-limited rural areas, cannot be screened. Under these circumstances, selective screening approach within the families suffering from thalassemia could be a viable approach in Bangladesh. In the study, anecdotal data (*n* = 605) on the family history of thalassemia suggest that 20% had another thalassemic siblings while 3% had two or more siblings with thalassemia. The majority of thalassemic couples in Bangladesh are identified retrospectively after diagnosis of one or more affected children: this could be used as a proxy indicator to test extended family to craft an effective carrier identification approach in Bangladesh. Given appropriate public health dissemination messages to affected and extended families, this could raise awareness of genetic susceptibility to thalassemia. In Sardinia, this approach was applied to only 15% of the adult population, which led to the detection of 90% of expected at-risk couples [47].

### Prenatal screening

A secondary prevention strategy emphasizes prenatal diagnosis followed by genetic counselling for the termination of pregnancy. The acceptability of the prenatal diagnosis and selective termination/abortion of an affected foetus is determined by many factors including religious, social and cultural backgrounds, personal experiences and beliefs. Therefore, ethical guidelines concerning genetic counseling, carrier screening and prenatal diagnosis need to be evaluated on the context of each society or country. Bangladesh is a predominantly Muslim country and social practices are heavily influenced by the religious practices. From the perspective of Islamic jurisprudence, it is permissible to perform abortion to protect mother’s life or health, or because of foetal anomaly which is incompatible with life [49]. A study conducted in the highly conservative Muslim society of Pakistan has shown that selective termination is accepted by affected parents irrespective of religious and social groups after genetic counseling [50]. Due to the extreme sensitivity of abortion from an Islamic viewpoint, mistakes are not permissible in the diagnosis of foetal anomalies [49].

Newborn screening (NBS) tests are practiced in developed countries using advanced molecular techniques to facilitate the early diagnosis of haemoglobinopathies for the prevention of complications and management [51]. Due to resource limitation in developing countries like Bangladesh, the application of NBS appears to be unrealistic.

The prime objective of a thalassemia prevention program is to educate the public as well as health professionals about the concept and consequence of genetic disorders. In a predominantly illiterate society/community, a special strategy is required to educate the public. General observation shows that health professionals are a vital source of information for a thalassemia affected family. To make an effective public awareness strategy, priority should be given to educate healthcare professionals first. Very little genetics is taught in undergraduate medical colleges in Bangladesh. At the postgraduate level, genetics tends to be a neglected specialty. Because of the young age structure of the population, thalassemia awareness focused on schools, colleges and universities had a significant impact in most developed societies and it would have the same impact in developing countries. It is essential to replace misunderstandings with correct information about the cause of genetic disease and resources available for its diagnosis, treatment and prevention. In this context, mass media such as television and newspaper could be vital tools for public awareness.

Listed below are some specific recommendations in the context of Bangladesh:

1. Virtually, nothing is known on various aspects including true burden, genetic spectrum, clinical outcome, morbidity and mortality of thalassemia in Bangladesh. Hence, research is the first and foremost priority to establish thalassemia as one of the major public health problems in Bangladesh.
2. Establish improved diagnostics and treatment facilities for thalassemias preferably in a one stop center.
3. Availability of genetic testing can prevent further birth of affected children in the family and can encourage for prenatal screening.
4. Prioritizing thalassemia awareness and access programs for the targeted population should be designed through innovative approaches.
5. Regional and international partnership and collaboration are necessary for the control and management of thalassemia in developing countries like Bangladesh.
6. Given the conservative religious society in Bangladesh, respected religious scholars should be included in the development of prevention policies (such as prenatal screening followed by abortion) on genetic disorders.
7. Electronic health information exchange (such as Apps) could be an important tool to ensure proper care of thalassemia patients. It is needed to focus on the understanding of the patient’s attribute to tailor intervention by leveraging information technology and patient-level data.
8. Private-public partnership must be promoted to tackle thalassemia in developing countries like Bangladesh.

## Conclusion

The present study depicts the overall treatment strategies prevailing in Bangladesh. We found a significant proportion of beta thalassemia carriers receiving transfusion. As mentioned previously, it could be due to misdiagnosis (e.g. concurrent iron/ vitamin deficiency) or a higher target Hb level for the patients due to lack of awareness among the practicing physicians. Beta thalassemia carriers can have mild anemia with Hb level ranging from 9 to 12 g/dL which does not warrant transfusion to normalize the Hb level. Individual response and adaptation to anemia may also play a role in selecting patients for transfusion. On the other hand, in case of HbE beta thalassemia, the most crucial issue is to determine transfusion dependence. Other neighboring countries like Sri Lanka may serve as a model in re-evaluating the transfusion practices in Bangladesh, as regards to thalassemia [52]. Modification of the existing candidate selection criteria for transfusion requirement, which is mostly based on Hb level currently practice in Bangladesh, will play a significant role in screening out patients that might benefit from other essential yet often neglected therapeutic interventions. Further investigations are necessary to understand the epidemiology, mutation spectrum, clinical course and treatment outcomes in this thalassemia prone country, which is undergoing demographic transition.

## Abbreviations

HbE: Hemoglobin E
HBV: Hepatitis B virus
HCV: Hepatitis C virus
NCD: No communicable disease
NTDT: Non-transfusion dependent thalassemia
TDT: Transfusion dependent thalassemia
TFH: Thalassemia Foundation Hospital
